# Driving Performance and Clinical Decision-Making

**DOI:** 10.1001/jamanetworkopen.2025.54479

**Published:** 2026-01-02

**Authors:** Valerie Danesh, Anthony D. McDonald

**Affiliations:** Center for Applied Health Research, Baylor Scott & White Research Institute, Dallas, Texas ; Department of Medicine, Baylor College of Medicine, Houston, Texas; College of Engineering, University of Wisconsin at Madison

Driving represents a unique intersection of everyday functional activity and complex contexts, from newly licensed adolescent drivers engaging in newfound freedom, to adults engaging in driving for work and caregiving, to older adults navigating community mobility. While initial licensure requirements and fleet management safety programs are closely tethered to evaluative strategies for new or professional drivers, less is known about driving performance in broad segments of driving populations, including older adults with and without mild cognitive impairment. Safety assessments of older adult driving are complicated by self-regulation including driving-related decision-making, limited longitudinal data, and complexity in the definition of aging.

In their study, Parihar et al,^1^ report on residential driving by community-dwelling older adults by collecting kinematic data (eg, acceleration, braking, and wayfinding) using unobtrusive in-vehicle sensors in participants’ personal vehicles. This naturalistic study design captures everyday driving performance, which, when paired with cognitive testing and neuroimaging biomarkers in a cohort of 220 older adults (≥65 years) collected over a 10-year period (2015-2024), offers insight into driving behaviors over time. These data suggest that driving decision-making may be associated with changes in white matter hyperintensities discernible by magnetic resonance imaging. In this cohort of cognitively normal older adults, these neuroimaging biomarkers were associated with changes in driving decision-making measured as fewer driving trips and fewer complex or new driving routes.

Driving data combined with high fidelity neuroimaging and longitudinal cognitive assessments spanning a decade for a cohort of more than 200 cognitively normal older adults contributes a multimodal dataset that is unique in the field. Beyond exploring characteristics associated with driving behaviors within the cohort,^1^ a key contribution is the foundational nature of establishing a trajectory of driving behaviors during normal aging for future disease-specific comparisons; the few participants lost to follow-up (4 participants) speaks to the deep level of rigor. Analysis of driving as a functional outcome by combining joint clinical and transportation data streams is still in its infancy with high impact implications for clinical decision-making in both times of sickness and times of health. Parihar et al^1^ are thoughtfully establishing baseline measures that can be used by many future disciplines and specialties.

The study^1^ anchors the definition of aging on small vessel vascular disease. This definition is far more precise than prior transportation science studies that define aging numerically. Beyond small vessel vascular disease and cognitive measures, there are physical aspects of aging including strength, range of motion, and vision, which play a role in clinical decision-making specific to driving. These aspects may warrant future consideration. Beyond these definitions, future work may benefit from the inclusion of standardized driving self-assessments including the driver skills inventory, and driver behavior questionnaire.^2,3^ These scales can be used to triangulate between existing driving safety and epidemiological literature lacking naturalistic kinematic data. Similarly validated socioeconomic measures for individual self-reporting (eg, Protocol for Responding to and Assessing Patients’ Assets, Risks, and Experiences) could further promote associations of aging, driving, and socioeconomic status.^4^

In addition to findings that highlight associations between white matter disease and driving decision-making, this cohort^1^ offers methodologic value in serving as control comparisons for disease-specific investigations in the future. Furthermore, these results are important to automotive manufacturers as a convergence of transportation science and aging research. There is a significant economic benefit to additional quality-adjusted driving years for older adult drivers and in prognosticating vehicle driving years at the population level. Extending driving years for older adults may be closely linked to emerging technology and vehicle systems that support their driving needs and cognitive status. Parihar et al^1^ provides a benchmark for exploring these driving needs and documenting their evolution over time.
